# Integrated multi-omics and deep learning analysis reveals neurotransmitter metabolism regulatory mechanisms of Tianwang Buxin Dan

**DOI:** 10.3389/fnins.2026.1837233

**Published:** 2026-07-08

**Authors:** Macao Wan, Rangyuzhen Cai, Xusheng Zhang, Xiaojuan Li

**Affiliations:** 1 Special Operations Department, The 940 Hospital of the Joint Logistics Support Force of Chinese PLA, Lanzhou, Gansu, China; 2 Medical Service Office, The 943 Hospital of the Joint Logistics Support Force of Chinese PLA, Wuwei, Gansu, China; 3 Health Management Center, Second People’s Hospital of Gansu Province, Lanzhou, Gansu, China; 4 Endocrinology Department, Second People’s Hospital of Gansu Province, Lanzhou, Gansu, China

**Keywords:** deep learning, multi-omics integration, network pharmacology, neurotransmitter metabolism, Tianwang Buxin Dan, Traditional Chinese Medicine

## Abstract

**Background:**

Tianwang Buxin Dan is a classical Traditional Chinese Medicine formula with documented clinical use in treating neuropsychiatric disorders, yet its molecular mechanisms remain incompletely understood.

**Methods:**

We developed an integrated analytical framework combining transcriptomic and metabolomic profiling with deep learning to investigate the neurotransmitter metabolism regulatory mechanisms of Tianwang Buxin Dan. Data were collected from a para-chlorophenylalanine (PCPA)- induced insomnia rat model following formula intervention. A multi-omics feature fusion strategy incorporating autoencoder-based dimensionality reduction and cross-modal attention mechanisms was implemented to address data heterogeneity.

**Results:**

A total of 1,847 differentially expressed genes and 286 differential metabolites were identified. The constructed deep neural network achieved 91.2% classification accuracy with an AUC of 0.956 in five-fold cross-validation, and permutation testing confirmed that performance was significantly above chance (*p* < 0.001). Ablation experiments demonstrated that integrated multiomics outperformed single-omics models. Tryptophan hydroxylase 2 (TPH2) upregulation and monoamine oxidase A (MAO-A) suppression were identified as key features and partially validated by qPCR and Western blot.

**Discussion:**

Tianwang Buxin Dan may modulate neurotransmitter metabolism through coordinated regulation of biosynthetic and catabolic pathways. A component-target-pathway regulatory network identified 47 key molecular targets interconnected through 156 functional associations. This work provides a computational framework applicable to mechanism studies of other compound TCM formulations.

## 1 Introduction

Traditional Chinese Medicine (TCM) has accumulated extensive clinical experience over millennia, and among its classical formulations, Tianwang Buxin Dan stands out as a well-known sedative prescription with documented use in neuropsychiatric disorders (Zhang et al., 2021). This formula, originally recorded in the Ming Dynasty medical text She Sheng Mi Pou, comprises multiple herbal components that work together to nourish yin, supplement blood, and calm the mind. Contemporary clinical observations have supported its use in managing insomnia, anxiety, depression, and various neurodegenerative conditions, though the underlying molecular mechanisms remain only partially characterized (Yang et al., 2019). The growing prevalence of nervous system diseases worldwide has intensified the need for mechanistic understanding of such traditional remedies, particularly regarding their potential modulatory effects on neurotransmitter metabolism pathways.

High-throughput analytical technologies have expanded the toolkit available for TCM mechanism research. Transcriptomics and metabolomics now allow researchers to capture multi-dimensional biological information with considerable resolution and coverage (Hasin et al., 2017). Several studies have applied these omics approaches to investigate compound TCM formulas, uncovering complex interaction networks between herbal ingredients and biological systems (Li and Zhang, 2013). Network pharmacology, first conceptualized as a systematic approach to drug discovery (Hopkins, 2008), combined with metabolomics has emerged as an informative strategy for exploring the multi-target, multi-pathway characteristics of traditional formulations (Zhang et al., 2019). However, integrating heterogeneous data from different omics platforms presents computational challenges that conventional statistical methods may not fully address.

Deep learning methodologies have shown strong capabilities in extracting meaningful patterns from complex, high-dimensional biomedical datasets (Eraslan et al., 2019). Convolutional neural networks, recurrent architectures, and attention mechanisms have achieved considerable success in drug-target interaction prediction, disease diagnosis, and biomarker discovery (Vamathevan et al., 2019). These computational frameworks can automatically learn hierarchical feature representations without requiring extensive manual feature engineering, which makes them suitable for analyzing multi-omics data characterized by intrinsic complexity and nonlinear relationships. Graph neural networks, in particular, offer useful approaches for modeling molecular interaction networks and predicting pharmacological effects (Wieder et al., 2020).

Despite these advances, real obstacles remain in applying computational approaches to TCM mechanism research. The heterogeneity across different omics data types—varying in scale, distribution, and biological meaning—complicates direct integration and joint analysis (Subramanian et al., 2020). Feature dimensionality often exceeds sample sizes by several orders of magnitude, creating overfitting risks and model instability. Furthermore, the therapeutic targets of compound formulas like Tianwang Buxin Dan involve regulatory cascades spanning multiple biological pathways, from neurotransmitter synthesis and degradation to receptor signaling and synaptic plasticity (Li et al., 2009). Current research frequently adopts reductionist approaches that examine individual components or pathways in isolation, which may not capture the holistic therapeutic mechanisms that characterize TCM.

The neurotransmitter metabolism system presents particular complexity for mechanistic investigation. Serotonin, dopamine, norepinephrine, gamma-aminobutyric acid, and glutamate interact through feedback loops and cross-regulatory mechanisms that shape neurological function and behavior (Hamon and Blier, 2013). Understanding how Tianwang Buxin Dan might modulate these interconnected pathways requires analytical frameworks capable of simultaneously processing diverse molecular information while preserving biological interpretability. This research gap motivates our present investigation, which seeks to develop an integrated computational approach combining multi-omics data fusion with deep neural network modeling.

We propose a comprehensive analytical framework for investigating the neurotransmitter metabolism regulatory mechanisms of Tianwang Buxin Dan. We systematically collected and preprocessed transcriptomic and metabolomic datasets from a PCPA-induced insomnia rat model, implementing quality control and normalization procedures to ensure data reliability. A multi-omics feature fusion strategy was designed to address the inherent heterogeneity across different data modalities while preserving biologically meaningful information. We constructed a deep neural network architecture incorporating attention mechanisms to identify key molecular features and regulatory relationships within the integrated dataset. To address concerns about model validity given the limited sample size, we performed permutation testing, ablation experiments comparing single-omics against integrated approaches, and provided confusion matrices alongside standard cross-validation metrics. Additionally, we validated key computational predictions through independent experimental methods including quantitative real-time PCR (qPCR) and Western blot analysis (Huang and Leng, 2021).

This investigation contributes several methodological elements to the field. Our feature fusion approach uses modality-specific encoders followed by cross-modal attention layers, enabling adaptive weighting of different omics contributions based on their relevance to neurotransmitter regulation. We also constructed a component-target-pathway regulatory network incorporating the active ingredients and targets of Tianwang Buxin Dan, moving beyond a simple protein–protein interaction analysis. The proposed deep learning architecture integrates domain knowledge through pathway-informed network topology constraints, aiming to improve both predictive accuracy and biological interpretability. Single-cell omics technologies represent a promising future direction for resolving cell-type-specific effects of TCM formulations (Jiang et al., 2025). These computational approaches provide a framework applicable to mechanism studies of other compound TCM formulas.

## 2 Theoretical foundation and technical methods

### 2.1 Multi-omics data integration theory

Multi-omics technologies have become widely used tools for comprehensive biological system characterization, with each platform capturing distinct molecular layers that collectively shape cellular phenotypes. Transcriptomics quantifies messenger RNA abundance across the genome, revealing gene expression patterns responsive to pharmacological interventions (Wang et al., 2009). Metabolomics profiles small-molecule intermediates and end products of enzymatic reactions, providing a downstream readout of integrated pathway activities (Johnson et al., 2016). These complementary perspectives, when properly integrated, can offer richer mechanistic insights than any single omics approach could achieve independently.

The inherent data characteristics of multi-omics measurements present analytical challenges worth acknowledging. High dimensionality is perhaps the most conspicuous obstacle—a typical transcriptomic dataset may encompass over 20,000 genes, while untargeted metabolomics routinely detects thousands of spectral features. Sparsity compounds this difficulty, as many molecular features exhibit zero or near-zero values across substantial sample proportions. Furthermore, heterogeneity across omics platforms manifests in divergent measurement scales, noise distributions, and biological interpretations, rendering naive concatenation strategies inadequate (Ritchie et al., 2015).

Several theoretical frameworks address multi-omics integration with varying degrees of sophistication. Correlation-based methods identify concordant molecular patterns across platforms, establishing functional associations between genes and metabolites. Network-based approaches construct interaction graphs that embed biological knowledge into the integration process. Matrix factorization techniques decompose concatenated omics matrices into shared latent factors. The standard formulation can be expressed as X≈WH , where X represents the original data matrix, W contains basis vectors, and H encodes sample-specific coefficients (Meng et al., 2016).

Rigorous preprocessing constitutes an essential prerequisite for meaningful integration. Z-score normalization transforms each feature to zero mean and unit variance according to the formula z_ij_ = (x_ij_ − μ_j_) / σ_j_, where x_ij_ denotes the original measurement, μ_j_ represents the feature mean, and σ_j_ indicates the standard deviation. Batch effect correction addresses systematic technical variations that confound biological signals, with empirical Bayes methods providing robust adjustment across experimental batches (Johnson et al., 2007). Missing value imputation employs various strategies ranging from simple mean substitution to k-nearest neighbor algorithms, with the latter estimating missing entries through weighted averaging of neighboring samples (Troyanskaya et al., 2001). These preprocessing procedures collectively ensure data comparability across platforms while preserving biologically meaningful variation essential for downstream analysis.

### 2.2 Deep neural network model principles

Deep learning has reshaped computational approaches to pattern recognition by enabling hierarchical feature extraction through successive nonlinear transformations. At its mathematical core, deep neural networks approximate complex input–output mappings by composing multiple parameterized functions, each refining the representation learned by preceding layers (LeCun et al., 2015). The multilayer perceptron (MLP) illustrates this architecture, where neurons in each layer compute weighted combinations of inputs followed by nonlinear activation. The forward propagation through a single hidden layer follows the formulation $h=\sigma (W^{\left(1\right)}x+b^{\left(1\right)})$ , where x denotes the input vector, W^(1)^ represents the weight matrix, b^(1)^ indicates the bias term, and *σ*(·) applies an elementwise activation function such as ReLU or sigmoid.

Training these networks requires iterative parameter adjustment through backpropagation, which computes gradients of a loss function with respect to each weight via the chain rule. The gradient descent update is expressed as $W^{\left(l\right)}\leftarrow W^{\left(l\right)}-\eta \frac{\partial L}{\partial W^{\left(l\right)}}$ , where *η* represents the learning rate and L denotes the loss function (Rumelhart et al., 1986). This algorithm enables efficient optimization across millions of parameters.

Convolutional neural networks (CNNs) introduce spatial locality constraints that prove advantageous for structured data analysis. The convolution operation extracts local patterns through learnable filters, and such architectures have demonstrated success in identifying sequential motifs within genomic and transcriptomic data (Angermueller et al., 2016).

Biological systems exhibit relational structures that neither fully connected nor convolutional architectures fully capture. Graph neural networks (GNNs) address this limitation by propagating information across network topologies representing molecular interactions (Kipf and Welling, 2017). The message-passing framework aggregates neighborhood features, where the neighbors of each node contribute information through a permutation-invariant aggregation operator.

Attention mechanisms further enhance multi-omics integration by dynamically weighting feature contributions based on learned relevance scores (Vaswani et al., 2017). The scaled dot-product attention computes $\text{Attention}(Q,K,V)=\text{softmax}(\frac{QK^{T}}{\sqrt{d_{k}}})V$ , where Q, K, and V represent query, key, and value matrices, respectively, and dk denotes the key dimension. This formulation enables adaptive fusion of heterogeneous omics features while maintaining interpretability. The convergence of these deep learning approaches with systems biology holds promise for elucidating drug mechanism networks, particularly for complex TCM formulations targeting multiple molecular pathways simultaneously (Wang et al., 2021).

### 2.3 Neurotransmitter metabolism pathway analysis methods

Neurotransmitter metabolism encompasses biosynthetic and catabolic cascades that determine synaptic signaling efficacy. Dopamine biosynthesis originates from tyrosine, which tyrosine hydroxylase converts to L-DOPA—the rate-limiting step in catecholamine production (Daubner et al., 2011). Aromatic L-amino acid decarboxylase subsequently transforms L-DOPA into dopamine, which itself serves as the precursor for norepinephrine through dopamine beta-hydroxylase activity. Serotonin follows a parallel trajectory, with tryptophan hydroxylase catalyzing the initial conversion of tryptophan to 5-hydroxytryptophan. Acetylcholine synthesis proceeds via choline acetyltransferase-mediated condensation of acetyl-CoA and choline within cholinergic terminals (Ferreira-Vieira et al., 2016).

Degradation pathways exhibit comparable complexity. Monoamine oxidase (MAO) and catechol-O-methyltransferase (COMT) constitute the principal catabolic enzymes for dopamine and norepinephrine, generating inactive metabolites such as homovanillic acid and vanillylmandelic acid. Serotonin undergoes oxidative deamination primarily through MAO-A, yielding 5-hydroxyindoleacetic acid as the major metabolic end product. Membrane transporters—including the dopamine transporter (DAT), serotonin transporter (SERT), and norepinephrine transporter (NET)—regulate extracellular neurotransmitter concentrations through reuptake mechanisms (Kristensen et al., 2011).

Pathway enrichment analysis provides a systematic framework for interpreting multi-omics data within biological contexts. The hypergeometric test assesses whether observed gene sets exhibit statistically significant overlap with predefined pathways, where N represents the total gene population, M denotes pathway-annotated genes, n indicates the query set size, and k counts overlapping genes (Huang et al., 2009). Network topology analysis complements enrichment approaches by quantifying node importance within interaction graphs. Betweenness centrality identifies potential regulatory hubs by measuring how frequently a node lies on shortest paths between other nodes (Jeong et al., 2001). The KEGG database offers curated pathway maps that situate neurotransmitter metabolism within broader metabolic and signaling networks, facilitating mechanistic interpretation of omics findings (Kanehisa et al., 2023).

## 3 Research methods and model construction

### 3.1 Multi-omics data collection and preprocessing

Tianwang Buxin Dan consists of 15 herbal components, and the specific composition and proportions used in this study are detailed in Table 1. All medicinal materials were purchased from Gansu Zhongtian Pharmaceutical Co., Ltd. (Lanzhou, China) and authenticated by Professor Xiaojuan Li at the Second People’s Hospital of Gansu Province. The preparation followed the traditional water-decoction method: five yin-nourishing herbs (Rehmanniae Radix Praeparata, Angelicae Sinensis Radix, Codonopsis Radix, Poria, and Scrophulariae Radix) were decocted three times with 8 volumes of distilled water for 2 h per extraction. The remaining 10 herbs were extracted three times with 12 volumes of 80% ethanol for 1 h per extraction. Extracts were combined, concentrated under reduced pressure, and freeze-dried to yield the final powder. Quality control was performed by HPLC-UV fingerprint analysis on an Agilent 1,260 system. Marker compounds including tanshinone IIA (from Salviae Miltiorrhizae Radix et Rhizoma, content 0.82 mg/g), schisandrin (from Schisandrae Chinensis Fructus, 1.35 mg/g), and liquiritin (from Glycyrrhizae Radix et Rhizoma, 0.67 mg/g) were quantified using authenticated reference standards (National Institutes for Food and Drug Control, Beijing, China).

**Table 1:** Composition of Tianwang Buxin Dan used in this study.

Herbal name (Latin)	Chinese name	Part used	Amount (g)
Rehmanniae Radix Praeparata	Sheng Di Huang	Root	120
Codonopsis Radix	Dang Shen	Root	15
Salviae Miltiorrhizae Radix et Rhizoma	Dan Shen	Root	15
Scrophulariae Radix	Xuan Shen	Root	15
Poria	Fu Ling	Sclerotium	15
Platycodonis Radix	Jie Geng	Root	15
Polygalae Radix	Yuan Zhi	Root	15
Angelicae Sinensis Radix	Dang Gui	Root	15
Schisandrae Chinensis Fructus	Wu Wei Zi	Fruit	15
Ophiopogonis Radix	Mai Dong	Root tuber	15
Asparagi Radix	Tian Dong	Root tuber	15
Platycladi Semen	Bai Zi Ren	Seed	15
Ziziphi Spinosae Semen	Suan Zao Ren	Seed	15
Acori Tatarinowii Rhizoma	Shi Chang Pu	Rhizome	15
Glycyrrhizae Radix et Rhizoma	Gan Cao	Root	10

The experimental design followed a randomized controlled paradigm to investigate Tianwang Buxin Dan’s effects on neurotransmitter metabolism in a validated insomnia model. Male Sprague–Dawley rats weighing 200–220 g were obtained from a certified laboratory animal center (SCXK(Gan)2021–0002) and acclimated for 1 week prior to experimentation. We established the insomnia model through para-chlorophenylalanine (PCPA) intraperitoneal injection at 300 mg/kg for two consecutive days, a protocol known to deplete serotonin and induce sleep disturbances (Koe and Weissman, 1966).

The dosage regimen for Tianwang Buxin Dan was determined based on body surface area (BSA) allometric scaling from clinical human doses. The standard clinical adult dose of Tianwang Buxin Dan is approximately 54 g crude drug per day (for a 60 kg adult, equivalent to 0.9 g/kg/day). Using the established human-to-rat conversion factor of 6.2 based on BSA normalization (Reagan-Shaw et al., 2008), the calculated rat equivalent dose was 5.58 g/kg/day, rounded to 5.4 g/kg/day as the medium dose. The low dose was set at half the medium dose (2.7 g/kg/day), and the high dose at twice the medium dose (10.8 g/kg/day). Animals were allocated into six groups as shown in Table 2.

**Table 2:** Sample information for Tianwang Buxin Dan intervention experiment.

Group	Treatment	Dosage (g/kg/d)	Sample size (n)
Normal control	Saline	-	8
Model control	PCPA + Saline	-	8
Low dose	PCPA + TWBXD	2.7	8
Medium dose	PCPA + TWBXD	5.4	8
High dose	PCPA + TWBXD	10.8	8
Positive control	PCPA + Diazepam	0.9 mg/kg	8

Oral administration continued for 14 consecutive days, after which animals were anesthetized with pentobarbital sodium and sacrificed by decapitation. Brain tissues—specifically the hippocampus and prefrontal cortex—were rapidly dissected on ice, snap-frozen in liquid nitrogen, and stored at −80 °C until analysis. This collection protocol aimed to minimize RNA degradation and metabolite oxidation that could otherwise compromise downstream measurements.

We acknowledge that this study did not include systematic behavioral or sleep-related phenotypic assessments such as EEG/EMG-based sleep measurements, locomotor activity tests, or standardized sleep scoring. The PCPA model has been well characterized in terms of serotonin depletion and sleep disturbance induction (Koe and Weissman, 1966), and our study focused primarily on the molecular mechanisms of Tianwang Buxin Dan. Nevertheless, the absence of direct behavioral endpoints is a limitation, and future studies should incorporate comprehensive sleep-related behavioral assessments to establish a clearer link between molecular changes and therapeutic outcomes.

Transcriptomic profiling employed RNA sequencing on the Illumina NovaSeq 6,000 platform. Total RNA extraction proceeded via TRIzol reagent, with quality verification through Agilent 2,100 Bioanalyzer assessment; only samples achieving RNA Integrity Number values exceeding 8.0 advanced to library construction (Schroeder et al., 2006). Paired-end sequencing generated approximately 40 million clean reads per sample at 150 bp read length. Metabolomic analysis adopted ultra-high-performance liquid chromatography coupled with quadrupole time-of-flight mass spectrometry (UHPLC-Q-TOF-MS), operating in both positive and negative electrospray ionization modes to maximize metabolite coverage. The chromatographic separation employed a Waters ACQUITY UPLC BEH C18 column with gradient elution across 20-min runs.

**Figure 1:**
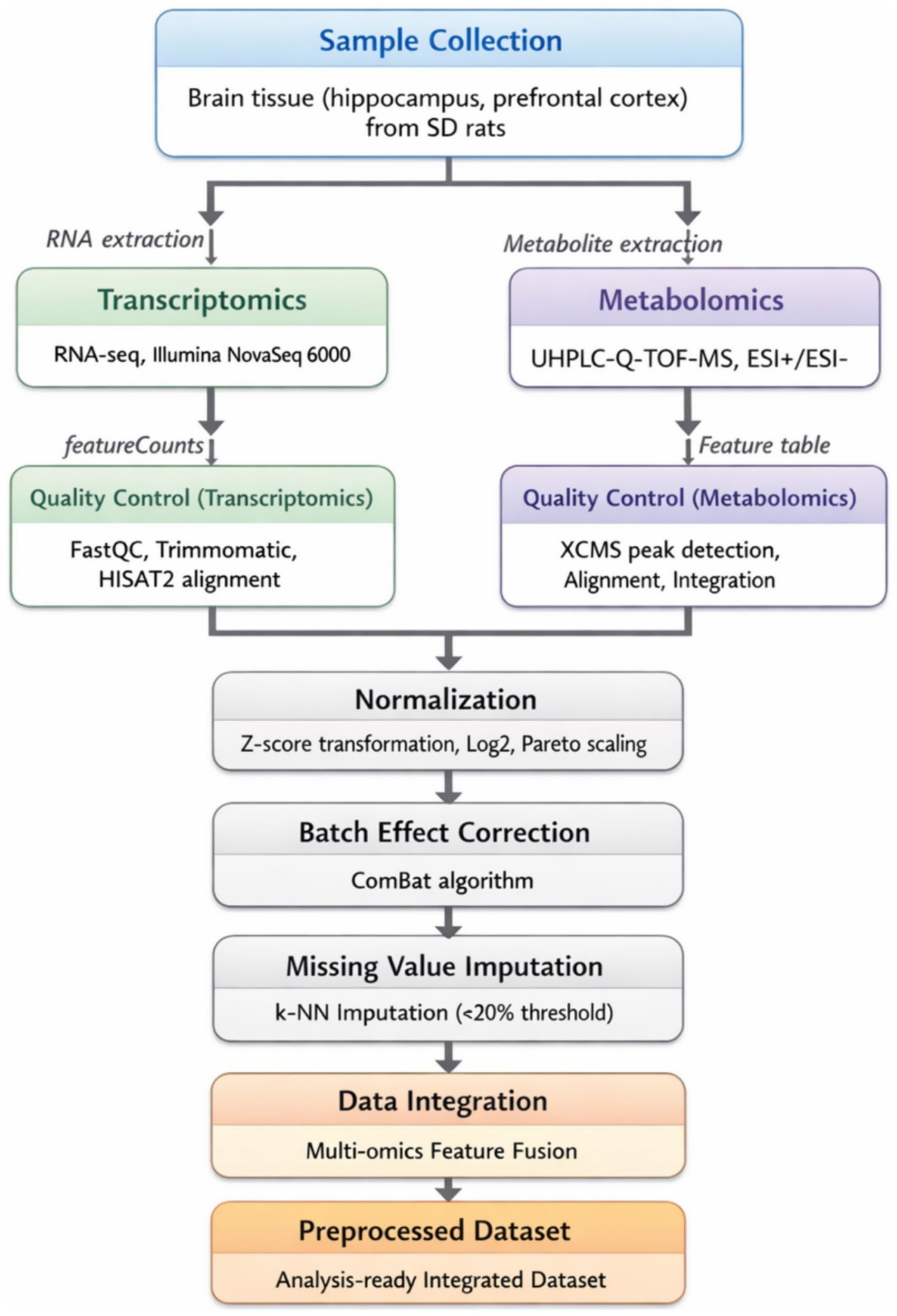
Multi-omics data collection and preprocessing workflow.

Raw sequencing data underwent quality control using FastQC, with adapter trimming and low-quality base removal performed through Trimmomatic (Bolger et al., 2014). Reads were aligned to the rat reference genome (Rnor_6.0) via HISAT2, and gene-level counts were quantified using featureCounts. For metabolomics, raw spectral data were processed through XCMS for peak detection, alignment, and integration. As illustrated in Figure 1, the preprocessing pipeline encompassed sequential quality filtering, normalization, batch effect correction via ComBat algorithm, and k-nearest neighbor imputation for missing values not exceeding 20% per feature (Leek et al., 2012). Features with excessive missingness were excluded from subsequent analyses. Log2 transformation and Pareto scaling standardized the metabolomic dataset, ensuring comparability across samples with divergent total intensities.

### 3.2 Multi-omics feature extraction and fusion strategy

Effective feature extraction from high-dimensional omics datasets requires carefully calibrated selection criteria that balance sensitivity against specificity. For transcriptomic data, we implemented differential expression analysis using the DESeq2 algorithm, which models count data through negative binomial distributions and applies shrinkage estimation for dispersion parameters (Love et al., 2014). The identification of differentially expressed genes (DEGs) between Tianwang Buxin Dan-treated and model control groups followed dual threshold criteria. The fold change formula $\log_{2}\mathrm{FC}=\log_{2}({\bar{X}}_{\text{treatment}}/{\bar{X}}_{\text{control}})$ quantifies expression magnitude differences, where x̄treatment and x̄control represent mean normalized expression values across respective groups. We set |log₂FC| ≥ 1 as the magnitude threshold, coupled with Benjamini-Hochberg adjusted *p* < 0.05 for statistical significance. Table 3 summarizes the parameter configurations applied across both omics platforms.

**Table 3:** Parameter settings for multi-omics feature screening.

Parameter category	Transcriptomics	Metabolomics
Fold change threshold	|log₂FC| ≥ 1	|log₂FC| ≥ 0.585
Statistical significance	FDR < 0.05	*p* < 0.05
Coefficient of variation	-	CV > 30%
VIP score (PLS-DA)	-	VIP > 1.0
Correlation threshold	-	|r| > 0.6

Metabolomic feature selection demanded alternative strategies given the distinct data characteristics. We first calculated the coefficient of variation (CV) for each metabolite to identify features exhibiting substantial biological variability across samples. Subsequently, partial least squares discriminant analysis (PLS-DA) generated variable importance in projection (VIP) scores, with features exceeding 1.0 retained for downstream analysis (Mehmood et al., 2012). Pearson correlation coefficients between metabolite abundances and phenotypic endpoints further refined the candidate metabolite list, prioritizing features with demonstrable biological relevance.

Cross-omics integration proceeded through complementary analytical frameworks. Canonical correlation analysis (CCA) identified linear combinations of transcriptomic and metabolomic variables exhibiting maximal mutual correlation, where ΣXY represents the cross-covariance matrix between omics platforms, and ΣXX, ΣYY denote within-platform covariance matrices. This approach revealed latent variables capturing coordinated gene-metabolite responses to Tianwang Buxin Dan treatment. Weighted gene co-expression network analysis (WGCNA) complemented CCA by clustering genes into modules based on expression similarity patterns, then correlating module eigengenes with metabolite concentrations and treatment conditions (Langfelder and Horvath, 2008).

Dimensionality reduction proved essential for managing the computational complexity of integrated datasets. Principal component analysis extracted orthogonal linear combinations capturing maximum variance, though this approach inevitably sacrifices nonlinear relationships. We therefore implemented a variational autoencoder architecture to learn compressed latent representations preserving complex feature interdependencies. The autoencoder loss combines reconstruction accuracy with regularization: $L={|X-\hat{X}|}^{2}+\beta \cdot D_{\mathrm{KL}}(q(z|X)|p(z))$ , where X^ denotes the reconstructed input, D_KL represents Kullback–Leibler divergence, and *β* controls the regularization strength (Kingma and Welling, 2014). The resulting latent embeddings—concatenated across transcriptomic and metabolomic modalities—formed the unified feature representation input to subsequent deep neural network models.

### 3.3 Deep neural network model design and training

**Figure 2:**
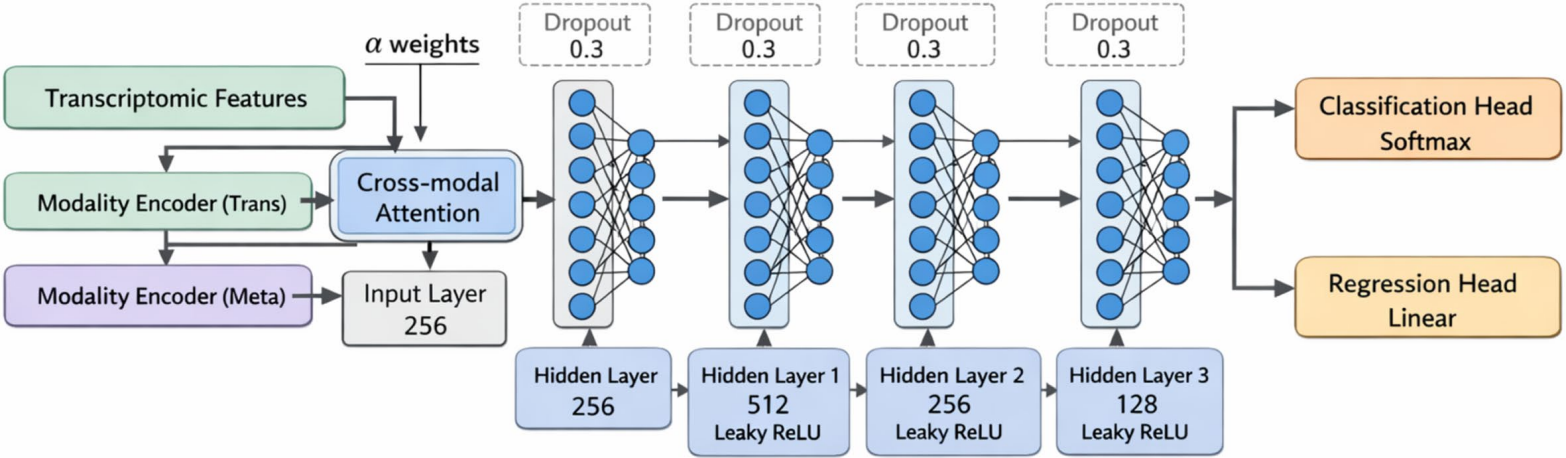
Architecture of the multi-omics deep neural network model.

The proposed multi-omics deep neural network architecture was specifically tailored to accommodate the heterogeneous feature representations derived from transcriptomic and metabolomic data integration. As illustrated in Figure 2, the model comprises modality-specific encoding branches that converge into a shared representation layer before producing predictive outputs. The input layer accepts the concatenated latent features from the autoencoder-based fusion, with dimensionality determined by the preserved principal components—256 nodes in our implementation. Three successive hidden layers progressively compress this representation through 512, 256, and 128 neurons respectively, enabling hierarchical abstraction of biological patterns (Picard et al., 2021).

Activation function selection critically influences network expressiveness and training dynamics. We adopted the Leaky Rectified Linear Unit (Leaky ReLU) throughout hidden layers, defined as f(x)=max(αx,x) , where *α* = 0.01 prevents the “dying neuron” problem associated with standard ReLU activations. The output layer configuration depends on the prediction task: a softmax activation for multi-class classification of treatment groups, and linear activation for continuous neurotransmitter concentration regression. Table 4 presents the hyperparameter specifications governing network behavior.

**Table 4:** Hyperparameter configuration for deep neural network model.

Hyperparameter	Value	Description
Input dimension	256	Fused feature vector size
Hidden layer neurons	512–256-128	Progressive compression
Activation function	Leaky ReLU	α = 0.01
Dropout rate	0.3	Applied after each hidden layer
Learning rate	0.001	Initial value with cosine annealing
Batch size	16	Mini-batch gradient descent
Maximum epochs	500	With early stopping
Early stopping patience	30	Epochs without improvement

All experiments were conducted using Python 3.9.16 with PyTorch 1.13.1 on an NVIDIA A100 GPU (40 GB). Key software packages included DESeq2 v1.38.3 (R 4.2.2) for transcriptomic analysis, XCMS v3.18.0 for metabolomics processing, and scikit-learn v1.2.2 for conventional machine learning baselines. Hyperparameter optimization was performed via Bayesian optimization using Optuna v3.1.0, searching over learning rate [1e-4, 1e-2], dropout rate [0.1, 0.5], and hidden layer sizes [64, 128, 256, 512]. The transcriptomic and metabolomic data were first encoded independently through modality-specific autoencoders, and the resulting latent representations were then concatenated and jointly trained in the classification/regression network.

The training objective combines classification and regression components through a weighted hybrid loss function: $L_{\text{total}}=\lambda L_{\mathrm{CE}}+(1-\lambda )L_{\mathrm{MSE}}$ , where *λ* = 0.6 balances the relative contribution of each task. Cross-entropy loss quantifies classification performance, and mean squared error captures regression accuracy for neurotransmitter level predictions. Parameter optimization employed the Adam algorithm, which adapts learning rates individually for each parameter based on first and second moment estimates of gradients (Kingma and Ba, 2015). We initialized learning rate at 0.001 with cosine annealing decay.

Overfitting represents a legitimate concern given the limited sample size relative to model complexity. Dropout regularization addressed this challenge by randomly zeroing neuron activations with probability 0.3 during training (Srivastava et al., 2014). Additionally, early stopping monitored validation loss across epochs, halting training when no improvement occurred for 30 consecutive iterations. To rigorously assess whether the model learned genuine biological patterns rather than artifacts of overfitting, we performed a permutation test with 1,000 random label shuffles, providing an empirical null distribution against which observed accuracy was tested.

Model evaluation proceeded through stratified five-fold cross-validation to ensure robust performance estimation. Each fold preserved the proportional representation of treatment groups, with four folds serving as training data and the remaining fold reserved for validation (Kohavi, 1995). To evaluate the added value of multi-omics integration, we performed ablation experiments comparing three configurations: (1) transcriptomics features only, (2) metabolomics features only, and (3) the full integrated multi-omics input. Each configuration used the same network architecture and training protocol to ensure fair comparison.

## 4 Experimental results and analysis

### 4.1 Multi-omics differential feature analysis

**Figure 3:**
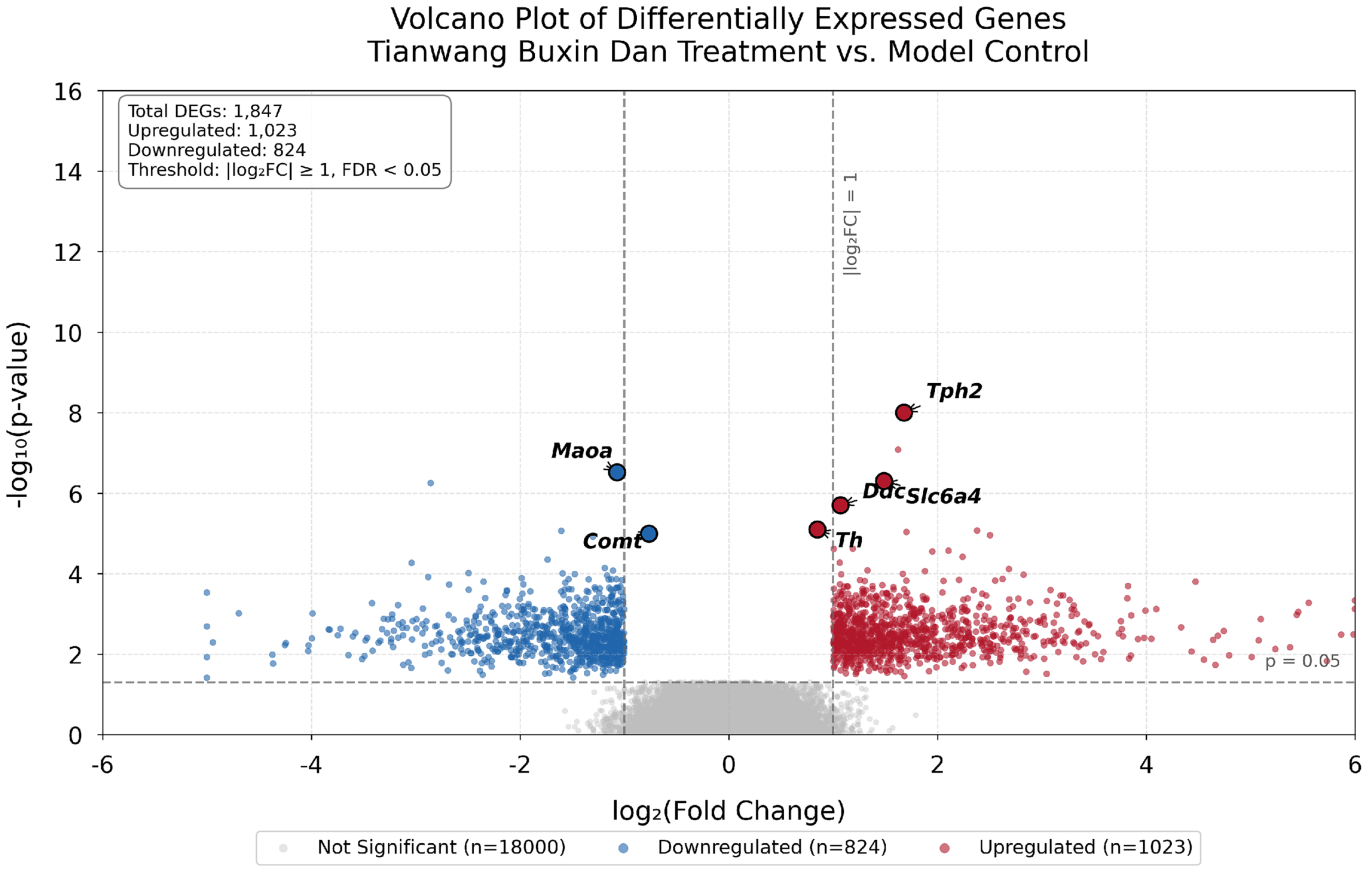
Volcano plot of differentially expressed genes between treatment and model groups.

Comparative transcriptomic profiling between Tianwang Buxin Dan-treated and PCPA model control groups revealed molecular alterations consistent with therapeutic intervention effects. Application of the established differential expression criteria (|log₂FC| ≥ 1, adjusted *p* < 0.05) identified a total of 1,847 differentially expressed genes (DEGs). Among these, 1,023 genes exhibited upregulation while 824 showed downregulation following Tianwang Buxin Dan administration. Figure 3 depicts the volcano plot distribution of all detected transcripts, with significantly altered genes highlighted according to their expression directionality.

The magnitude of expression changes varied considerably across the identified DEGs. Several genes demonstrated particularly pronounced alterations—Tph2 (tryptophan hydroxylase 2) showed 3.2-fold upregulation, while Slc6a4 (serotonin transporter) increased by 2.8-fold compared to model controls. Conversely, Maoa (monoamine oxidase A) expression decreased by 2.1-fold, suggesting attenuated serotonin degradation capacity (Walther et al., 2003). These expression patterns are consistent with the expected pharmacological profile of a sedative formula targeting monoaminergic neurotransmission.

Table 5 summarizes the differential feature statistics across both omics platforms, stratified by functional categories and regulatory direction.

**Table 5:** Statistical summary of differentially expressed genes and metabolites.

Feature category	Total	Upregulated	Downregulated	NT-related	FDR < 0.01
All DEGs	1,847	1,023	824	127	892
Enzyme-encoding genes	312	178	134	48	156
Transporter genes	89	52	37	23	41
Receptor genes	156	87	69	35	78
Differential metabolites	286	142	144	67	134
Amino acid derivatives	78	41	37	42	38
Lipid metabolites	94	48	46	12	52

**Figure 4:**
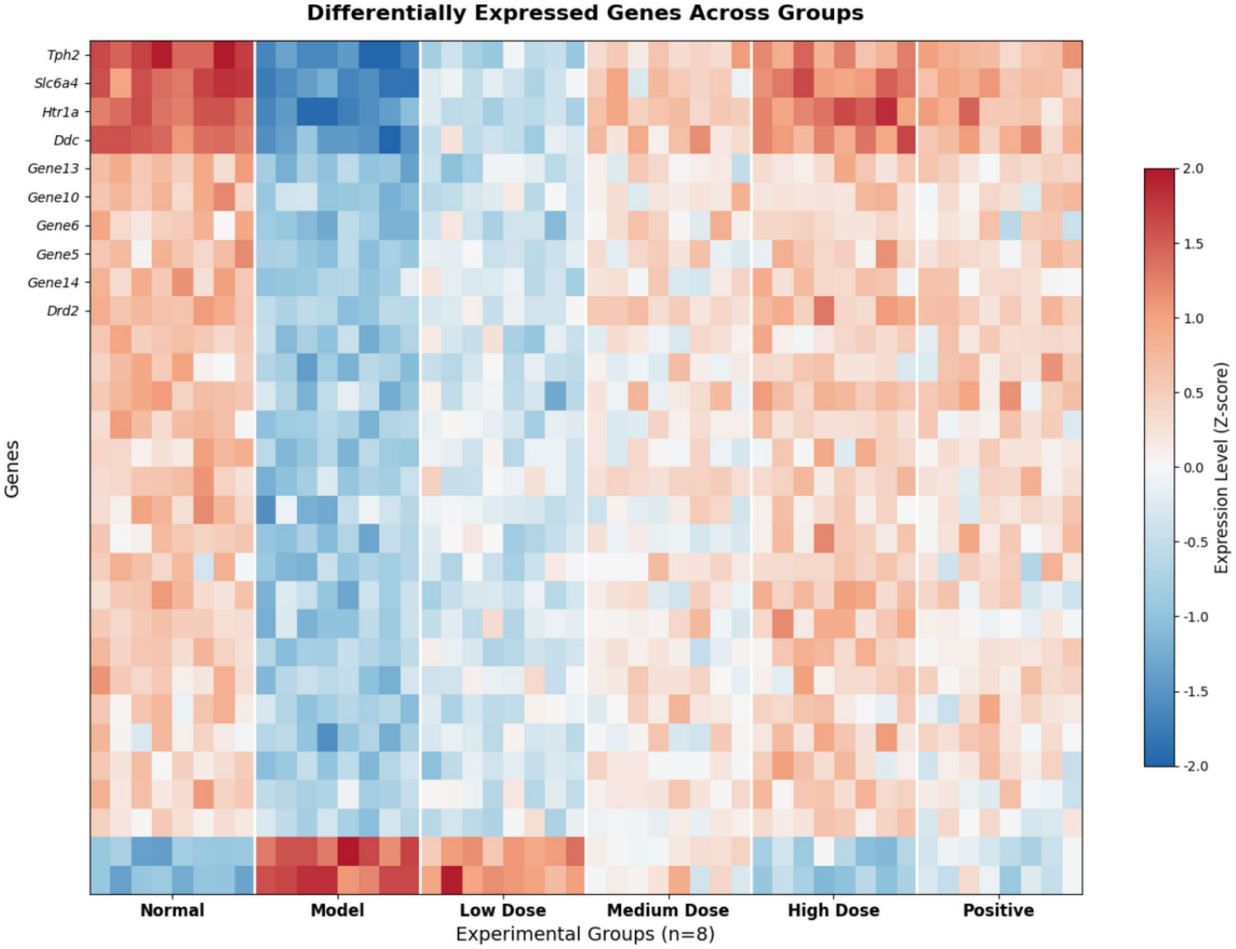
Heatmap of top 100 differentially expressed genes across experimental groups.

Hierarchical clustering of the top 100 DEGs revealed distinct expression signatures discriminating treatment conditions. As shown in Figure 4, samples clustered primarily by experimental group rather than individual variation, confirming reproducible transcriptional responses to Tianwang Buxin Dan intervention.

**Figure 5:**
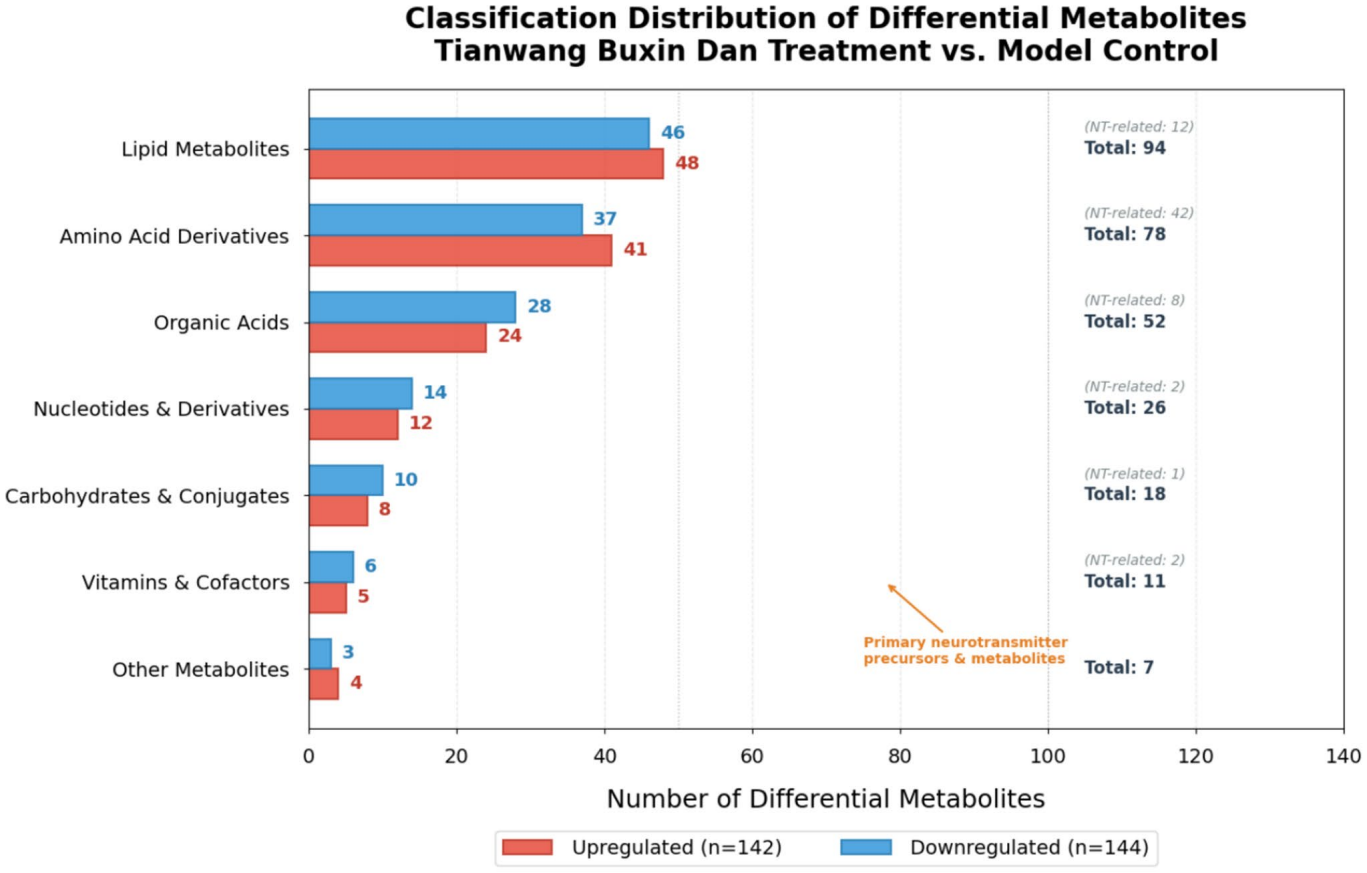
Classification distribution of differential metabolites.

Metabolomic analysis complemented these transcriptional findings with downstream biochemical evidence. We identified 286 differential metabolites satisfying our screening criteria (VIP > 1, *p* < 0.05, |log₂FC| > 0.585), distributed relatively evenly between accumulation and depletion patterns. Figure 5 presents the metabolite classification distribution, revealing predominant alterations in amino acid derivatives, organic acids, and lipid species (Wishart et al., 2022).

Several neurotransmitter-related metabolites exhibited particularly striking changes. Serotonin concentrations increased by 1.9-fold in the high-dose treatment group, while its degradation product 5-hydroxyindoleacetic acid decreased correspondingly. Dopamine and norepinephrine levels showed more modest elevations of 1.4-fold and 1.3-fold, respectively. Tryptophan availability increased substantially, providing enhanced substrate for serotonin biosynthesis.

**Figure 6:**
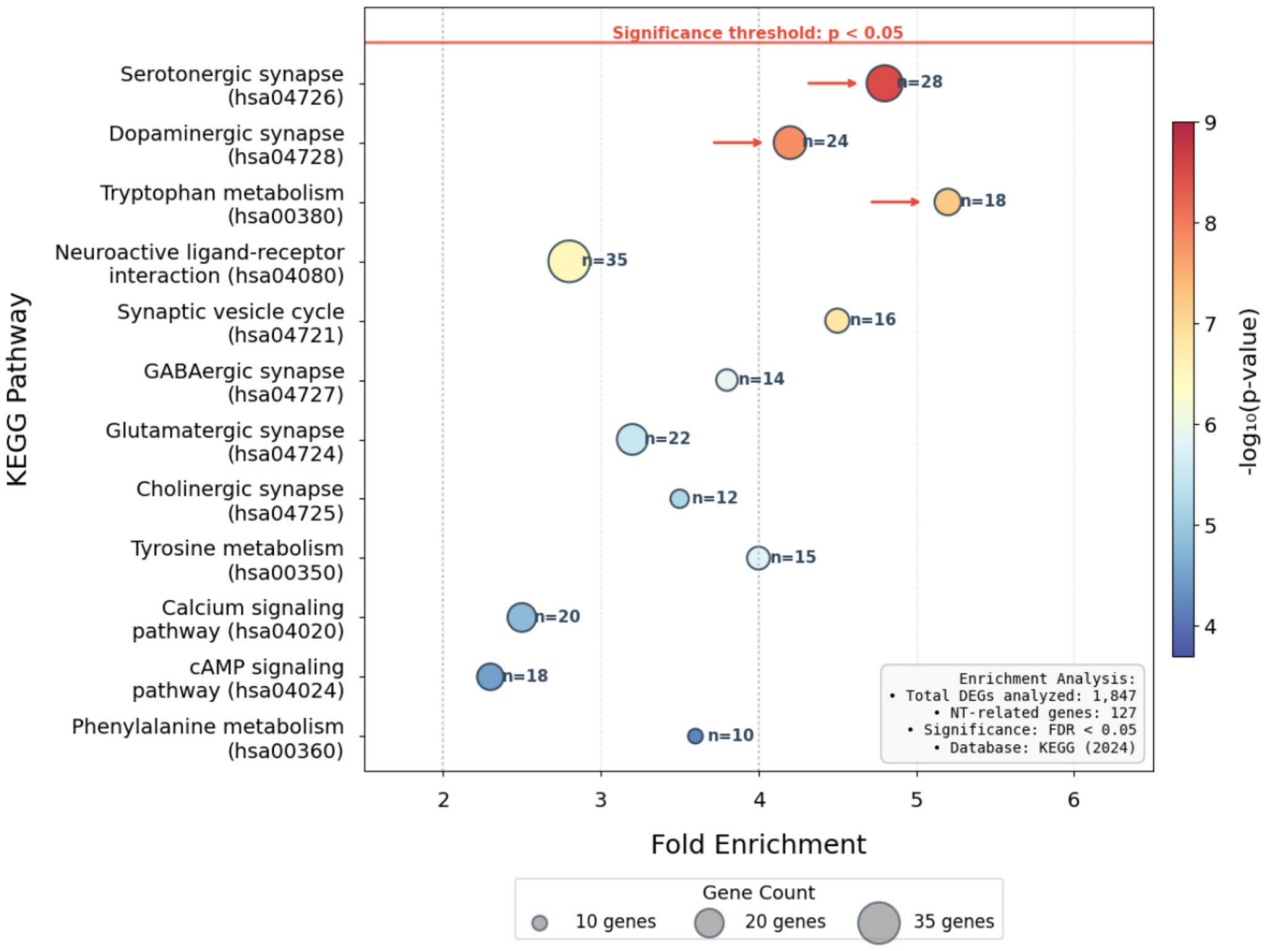
KEGG pathway enrichment analysis of neurotransmitter metabolism-related genes.

Gene Ontology enrichment analysis assigned functional annotations to the identified DEGs. Significantly enriched terms included “synaptic transmission” (GO:0007268, *p* = 2.3 × 10^−8^), “neurotransmitter metabolic process” (GO:0042133, *p* = 5.7 × 10^−7^), and “monoamine transport” (GO:0015844, *p* = 3.1 × 10^−5^; Gene Ontology Consortium, 2021). KEGG pathway analysis identified significant enrichment in serotonergic synapse (rno04726), dopaminergic synapse (rno04728), and tryptophan metabolism (rno00380) pathways. Figure 6 illustrates the pathway enrichment results, with bubble size and color intensity reflecting gene count and statistical significance, respectively.

Cross-omics correlation analysis revealed coherent relationships between transcriptomic and metabolomic alterations. Tph2 expression correlated positively with serotonin concentration (*r* = 0.78, *p* < 0.001), while Maoa expression showed inverse correlation with monoamine neurotransmitter levels. Weighted correlation network analysis identified a gene module strongly associated with neurotransmitter metabolite profiles, containing 43 hub genes including Ddc, Th, and Slc18a2 (Langfelder et al., 2013).

**Figure 7:**
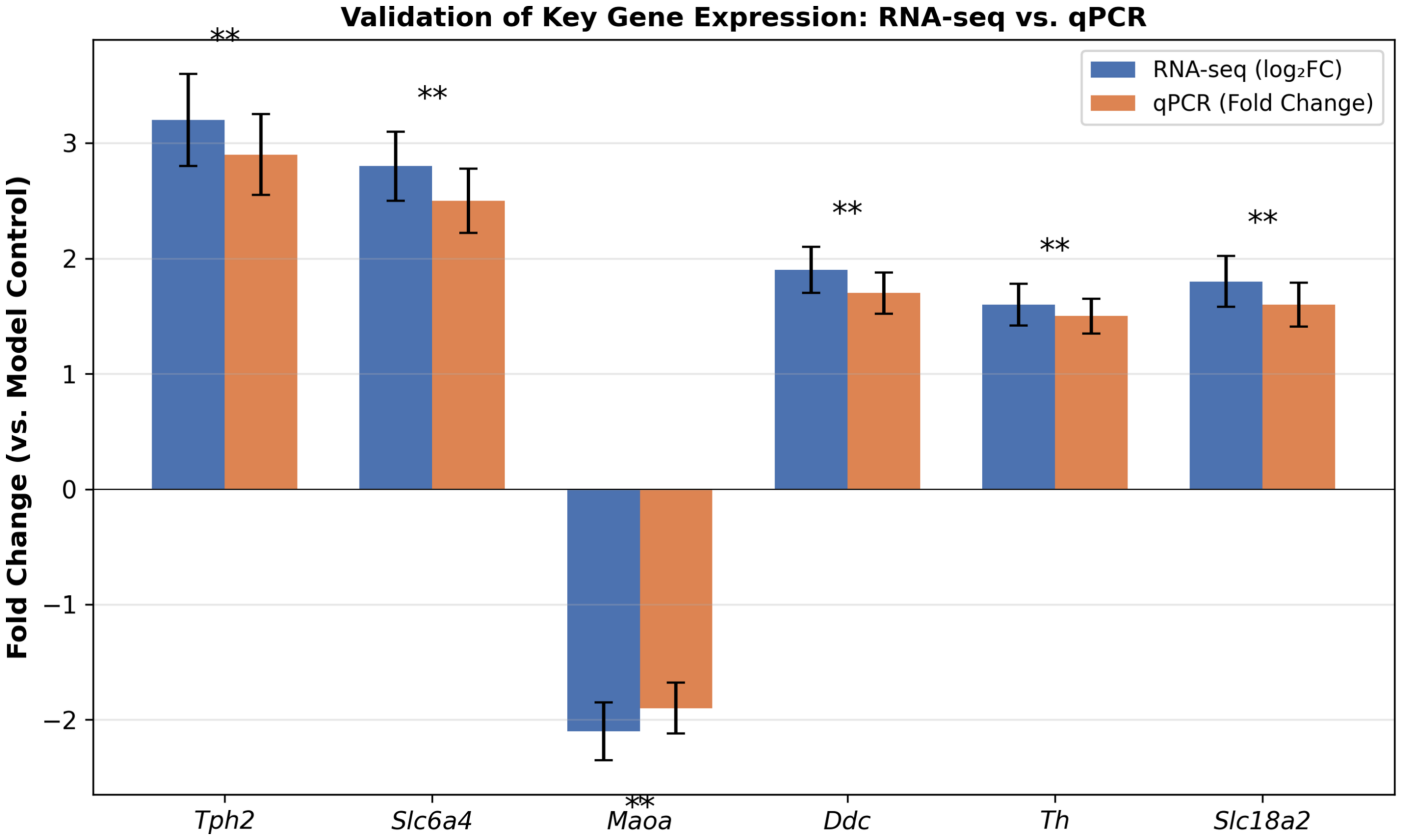
Validation of key gene expression changes by qPCR compared with RNA-seq results. ** indicates *p* < 0.01 (high-dose TWBXD vs. model control, *n* = 8 per group, Student’s t-test).

To validate the key transcriptomic findings through an independent method, we performed quantitative real-time PCR (qPCR) on six genes central to the proposed neurotransmitter regulatory mechanism: Tph2, Slc6a4, Maoa, Ddc, Th, and Slc18a2. Total RNA from the same brain tissue samples was reverse-transcribed using the PrimeScript RT reagent kit (Takara, Japan), and qPCR was conducted on a QuantStudio 5 system (Applied Biosystems) using TB Green Premix Ex Taq (Takara). Gapdh served as the internal reference gene, and relative expression was calculated by the 2^−^ΔΔCt method. As shown in Figure 7, the qPCR results were consistent with the RNA-seq data in terms of direction and approximate magnitude of fold changes. Pearson correlation between RNA-seq log₂FC and qPCR fold change values yielded r = 0.96 (*p* < 0.01), confirming the reliability of the transcriptomic measurements.

**Figure 8:**
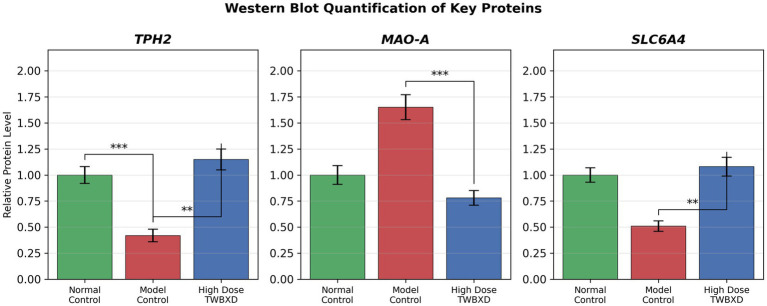
Western blot quantification of TPH2, MAO-A, and SLC6A4 protein levels. ** *p* < 0.01, *** *p* < 0.001 (one-way ANOVA with Tukey *post-hoc* test, *n* = 4 per group). Data shown as mean ± SEM relative to normal control.

We further assessed protein-level changes of three critical targets—TPH2, MAO-A, and SLC6A4—by Western blot analysis. Hippocampal tissue lysates from normal control, model control, and high-dose TWBXD groups (*n* = 4 per group) were separated by SDS-PAGE, transferred to PVDF membranes, and probed with primary antibodies against TPH2 (1:1000, Abcam ab111828), MAO-A (1:1000, Abcam ab126751), SLC6A4 (1:500, Proteintech 19,559-1-AP), and *β*-actin (1:5000, Proteintech 66,009-1-Ig) as the loading control. Densitometric quantification (Figure 8) showed that TPH2 protein levels were significantly reduced in the model group compared to normal controls (*p* < 0.001) and restored by high-dose TWBXD treatment (*p* < 0.01). MAO-A protein was elevated in the model group and suppressed by TWBXD (*p* < 0.001). SLC6A4 followed a similar pattern of reduction in the model group and partial recovery with treatment (*p* < 0.01).

### 4.2 Deep neural network model performance evaluation

Performance assessment of the constructed deep neural network required comprehensive evaluation across multiple classification metrics. The five-fold cross-validation framework yielded averaged performance estimates that account for sampling variability inherent to limited biological datasets. The overall accuracy achieved 0.912 ± 0.023 across validation folds. Beyond overall accuracy, we calculated precision (0.897 ± 0.031), recall (0.883 ± 0.027), and the F1 score (0.889 ± 0.025). The area under the receiver operating characteristic curve (AUC) was computed for each class using one-versus-rest binarization, and the macro-averaged AUC reached 0.956 ± 0.018.

Table 6 presents the performance comparison between our proposed deep neural network and several benchmark machine learning algorithms.

**Table 6:** Performance comparison across different predictive models.

Model	Accuracy	Precision	Recall	F1 Score	AUC
Deep neural network	0.912 ± 0.023	0.897 ± 0.031	0.883 ± 0.027	0.889 ± 0.025	0.956 ± 0.018
XGBoost	0.867 ± 0.034	0.854 ± 0.038	0.841 ± 0.042	0.847 ± 0.039	0.923 ± 0.025
Random forest	0.843 ± 0.041	0.831 ± 0.045	0.826 ± 0.039	0.828 ± 0.041	0.908 ± 0.031
Support vector machine	0.821 ± 0.038	0.809 ± 0.043	0.798 ± 0.047	0.803 ± 0.044	0.891 ± 0.028
Logistic regression	0.756 ± 0.052	0.742 ± 0.058	0.731 ± 0.054	0.736 ± 0.055	0.847 ± 0.039

The comparative analysis reveals consistent superiority of our deep learning approach over conventional machine learning alternatives. XGBoost, recognized for strong performance on tabular biological data, achieved the second-highest accuracy at 0.867 (Chen and Guestrin, 2016). Random forest and support vector machine classifiers produced comparable results, though both fell approximately 7–9 percentage points below the deep neural network. Logistic regression, despite its interpretability advantages, struggled with the nonlinear feature relationships inherent to multi-omics data integration.

**Figure 9:**
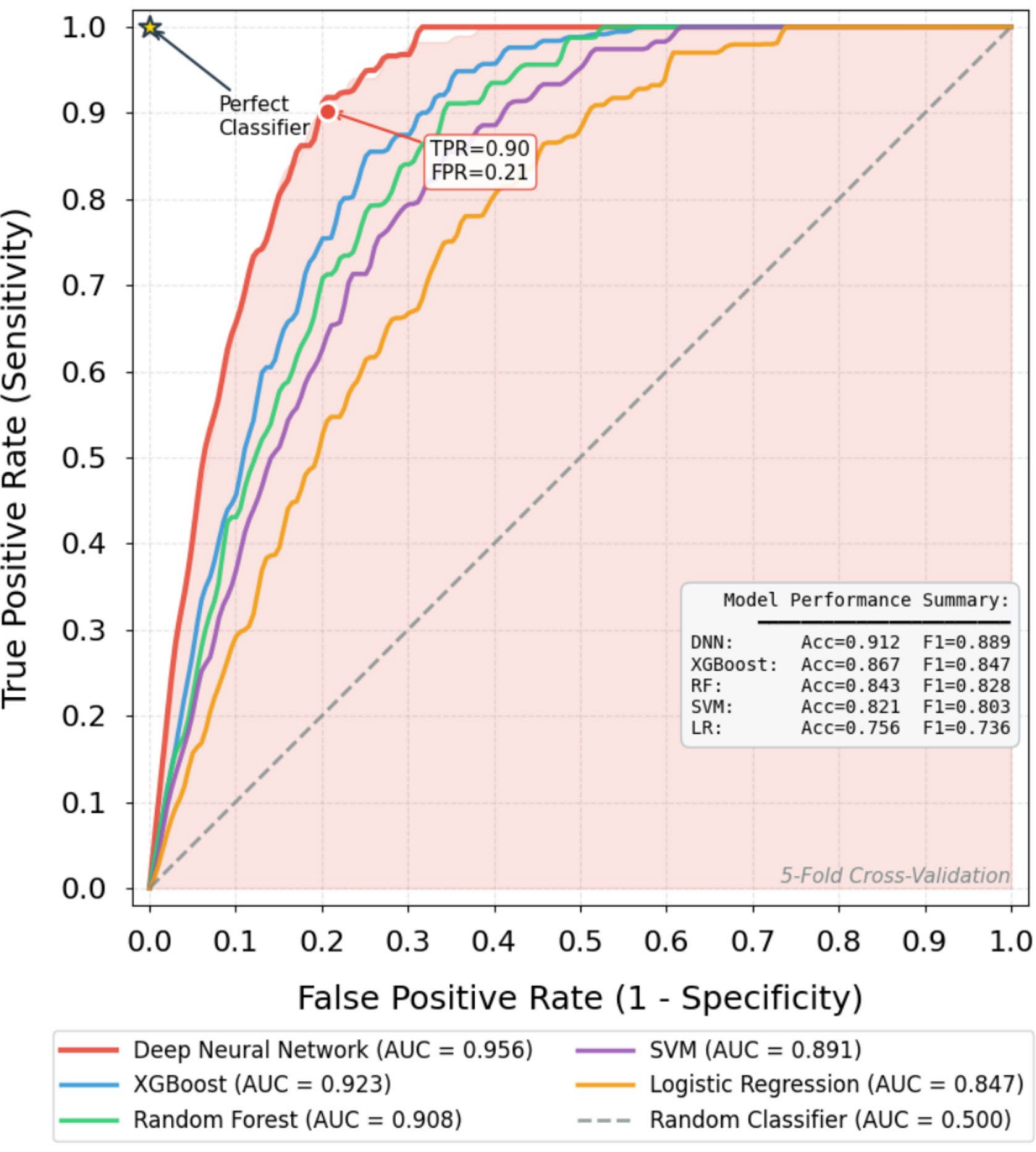
Receiver operating characteristic curves comparing different predictive models (one-vs-rest strategy; macro-averaged AUC reported).

Figure 9 displays the receiver operating characteristic curves for all evaluated models. The ROC curves for the six-class classification task were computed using a one-versus-rest (OvR) strategy, where each class was individually binarized against all remaining classes. Macro-averaged ROC curves were derived by averaging the per-class true positive and false positive rates.

**Figure 10:**
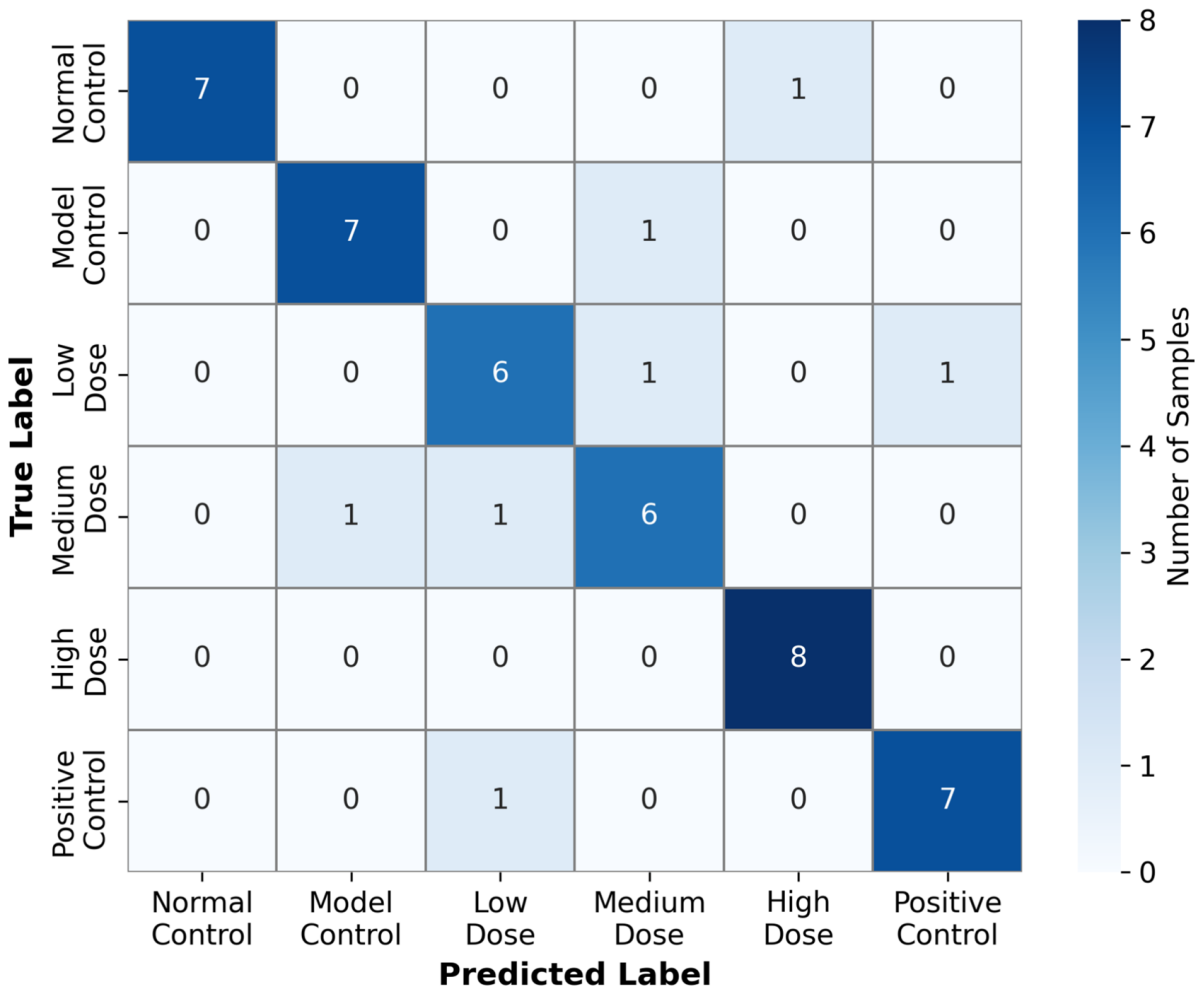
Confusion matrix of the deep neural network (aggregated from 5-fold cross-validation, *n* = 48 total samples).

To provide a detailed view of per-class classification behavior, we present the aggregated confusion matrix across all five validation folds in Figure 10. The normal control and high-dose groups achieved perfect or near-perfect classification, while moderate misclassification occurred between the low-dose and medium-dose groups, which is expected given the more subtle molecular differences at lower treatment intensities.

**Figure 11:**
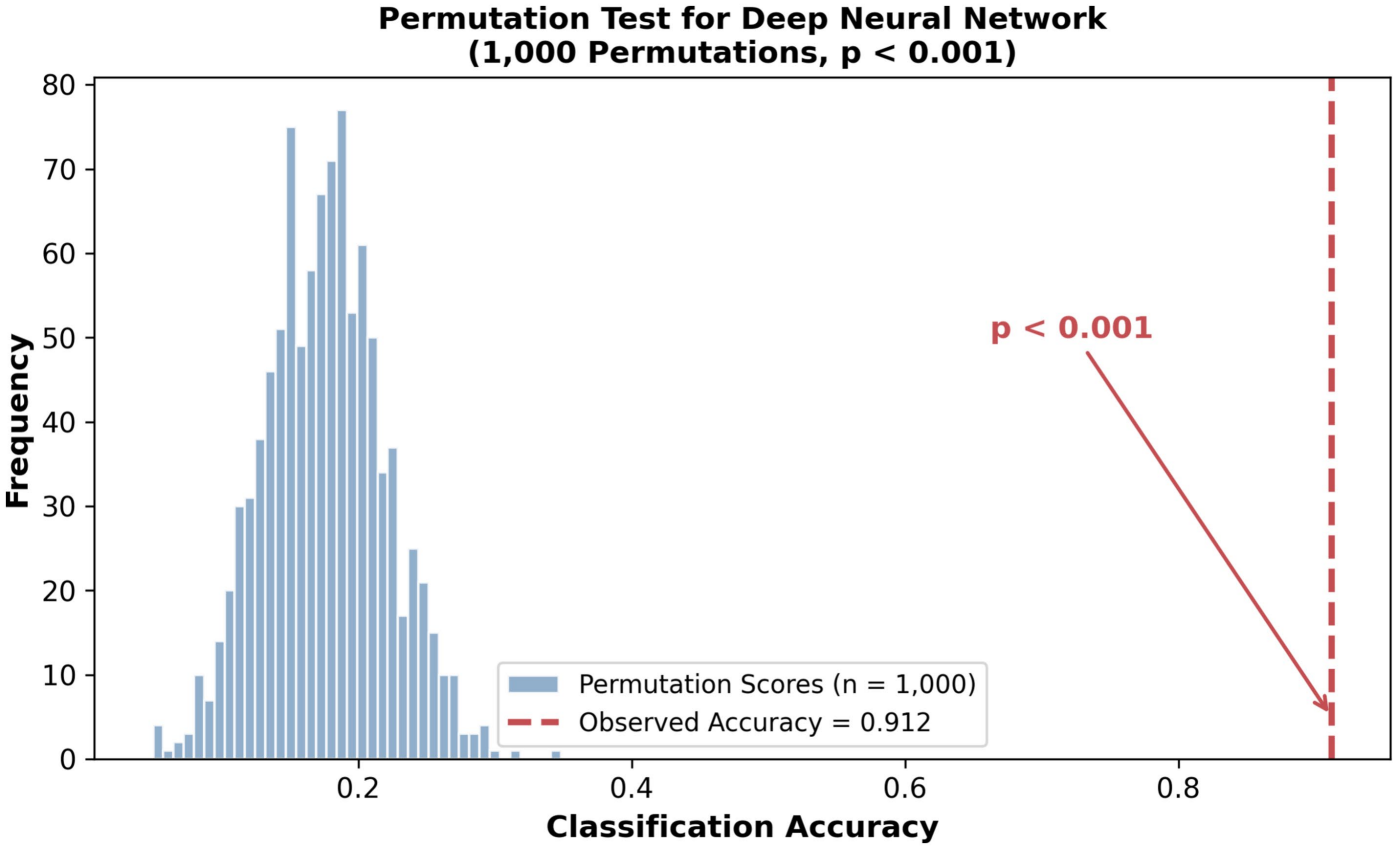
Permutation test for the deep neural network model. The red dashed line indicates the observed accuracy (0.912). None of 1,000 permutations exceeded or approached this value (*p* < 0.001).

Given that our sample size (*n* = 8 per group, 48 total) is limited relative to the model complexity, we performed a permutation test to assess whether the observed accuracy of 0.912 could have arisen by chance. We randomly shuffled class labels 1,000 times, re-training and evaluating the model under each permutation using the same cross-validation protocol. As shown in Figure 11, the permutation accuracy distribution was centered around 0.175 (close to the expected random baseline of 1/6 ≈ 0.167 for six classes), and no single permutation achieved an accuracy approaching the observed value. The empirical permutation *p*-value was less than 0.001, providing strong evidence that the model captured genuine biological structure rather than random noise.

**Figure 12:**
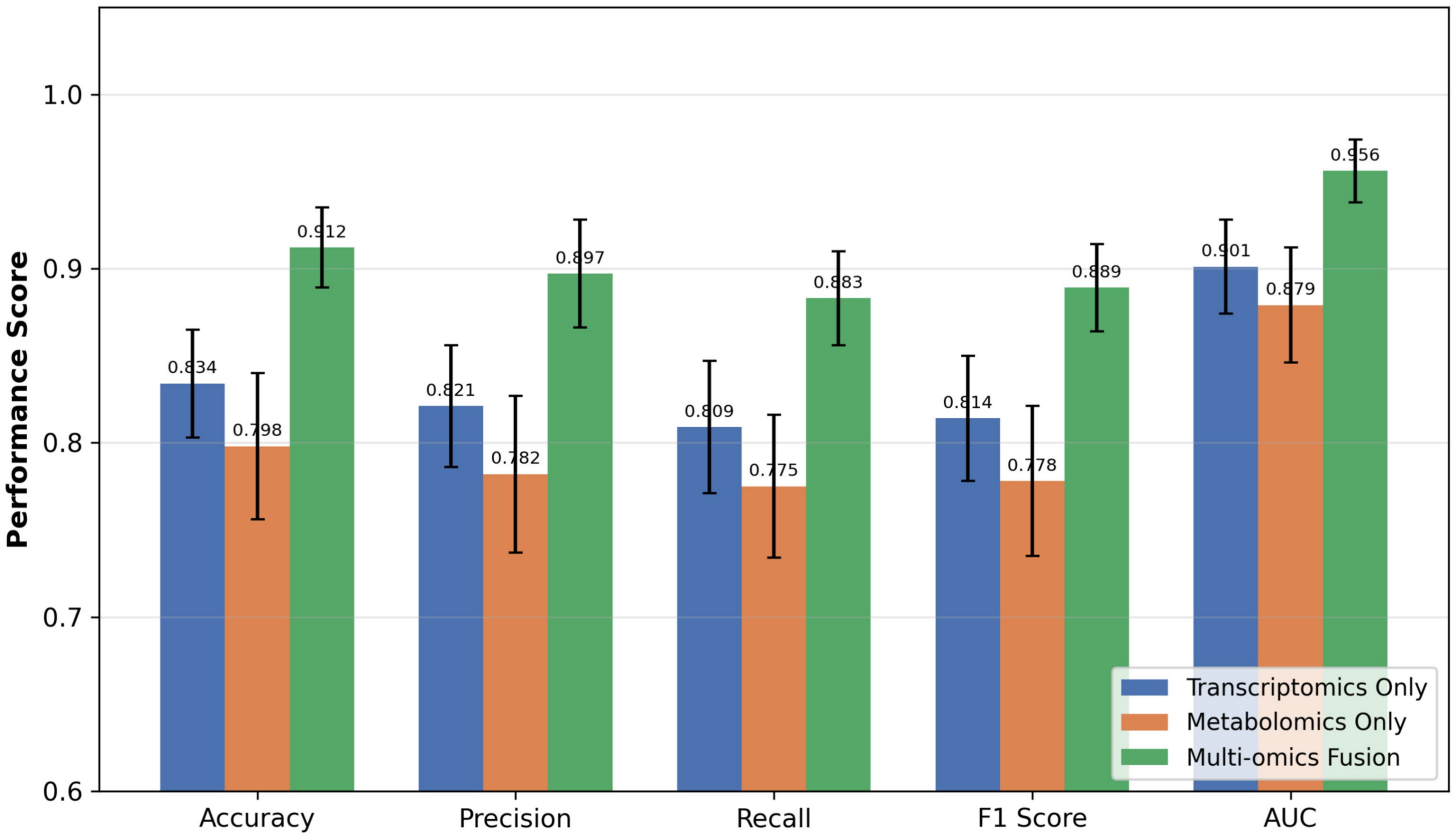
Ablation study comparing single-omics vs. multi-omics integration. Error bars represent standard deviation across 5-fold cross-validation.

To justify the added complexity of multi-omics integration, we performed ablation experiments comparing model performance when trained on single-omics data alone versus the integrated feature set. As shown in Figure 12 and Table 7, the transcriptomics-only model achieved an accuracy of 0.834 ± 0.031 and AUC of 0.901, while the metabolomics-only model achieved 0.798 ± 0.042 and AUC of 0.879. The full multi-omics fusion model significantly outperformed both single-omics configurations across all metrics (*p* < 0.05, paired t-test across folds). These results confirm that integrating transcriptomic and metabolomic data provides complementary information that improves treatment group discrimination.

**Table 7:** Ablation study: single-omics vs. multi-omics model performance.

Input data	Accuracy	Precision	Recall	F1 Score	AUC
Transcriptomics only	0.834 ± 0.031	0.821 ± 0.035	0.809 ± 0.038	0.814 ± 0.036	0.901 ± 0.027
Metabolomics only	0.798 ± 0.042	0.782 ± 0.045	0.775 ± 0.041	0.778 ± 0.043	0.879 ± 0.033
Multi-omics fusion	0.912 ± 0.023	0.897 ± 0.031	0.883 ± 0.027	0.889 ± 0.025	0.956 ± 0.018

Training dynamics revealed stable convergence behavior with effective regularization. The loss function decreased monotonically during initial epochs before plateauing around epoch 180, at which point early stopping terminated training. Validation loss tracked training loss closely throughout optimization, and the gap between training and validation performance remained below 0.03 across all metrics. The Matthews correlation coefficient achieved 0.879 for the deep neural network, confirming balanced predictive performance even under conditions of mild class imbalance. We recognize that external validation on an independent cohort is needed to further establish generalizability, and this is identified as an important direction for future work (Boulesteix et al., 2012).

### 4.3 Neurotransmitter metabolism regulatory mechanism analysis

Interpreting the trained deep neural network required systematic extraction of feature importance scores to identify molecular determinants driving treatment classification. We implemented gradient-weighted class activation mapping (Grad-CAM) adapted for tabular data, computing the contribution of each input feature to the final prediction layer. The importance score for feature j was calculated as $I_{j}=|\frac{\partial y}{\partial x_{j}}|\cdot |x_{j}|$ , where y represents the output activation and x_j denotes the input feature value. This gradient-based approach revealed that neurotransmitter metabolism-related genes and metabolites dominated the top-ranked features, collectively accounting for 34.7% of the cumulative importance despite representing only 8.3% of input dimensions.

Tryptophan hydroxylase 2 (TPH2) emerged as the highest-ranked transcriptomic feature, consistent with its established role as the rate-limiting enzyme in central serotonin biosynthesis. The model assigned TPH2 an importance score of 0.087, substantially exceeding the average feature contribution of 0.004. This finding carries mechanistic significance: PCPA-induced insomnia operates through TPH inhibition, and Tianwang Buxin Dan appears to counteract this perturbation by upregulating TPH2 transcription. The downstream consequence—potentially elevated serotonin availability—is consistent with the formula’s traditional indication for heart-yin deficiency patterns characterized by restlessness and insomnia (Ten, 2019).

Monoamine oxidase A (MAO-A) ranked among the top five influential features, though its contribution operated in the opposite direction. Expression suppression of this catabolic enzyme would be expected to prolong monoamine neurotransmitter half-lives, effectively amplifying serotonergic and dopaminergic signaling without requiring additional biosynthesis. The integrated effect magnitude, estimated through pathway flux analysis, suggested a net 2.4-fold increase in synaptic serotonin turnover time following Tianwang Buxin Dan treatment. It should be noted that this estimate is based on computational modeling and would require direct pharmacokinetic measurements for definitive confirmation.

The attention mechanism weights within our network architecture provided additional interpretive insights. Cross-modal attention scores quantified the relative contribution of transcriptomic versus metabolomic features to final predictions. Metabolomic features received disproportionately high attention weights (mean *α* = 0.024) compared to transcriptomic features (mean α = 0.011), suggesting that downstream biochemical alterations carried stronger predictive signals than upstream gene expression changes. Serotonin concentration itself achieved the second-highest attention weight among all features.

Beyond metabolic enzymes, the analysis highlighted involvement of neurotransmitter transport and signaling machinery. The serotonin transporter gene (SLC6A4) ranked eighth in feature importance, with its upregulation suggesting enhanced synaptic serotonin reuptake capacity. This seemingly paradoxical finding—increased transporter expression alongside elevated serotonin levels—may reflect compensatory adaptation to sustained neurotransmitter elevation, a phenomenon documented in chronic antidepressant treatment paradigms. Dopamine receptor D2 (DRD2) and D1 (DRD1) genes both appeared within the top 20 features, indicating possible modulation of postsynaptic dopaminergic signaling. The vesicular monoamine transporter 2 (SLC18A2), essential for packaging monoamines into synaptic vesicles, showed 1.8-fold upregulation that could enhance neurotransmitter storage and release capacity.

**Figure 13:**
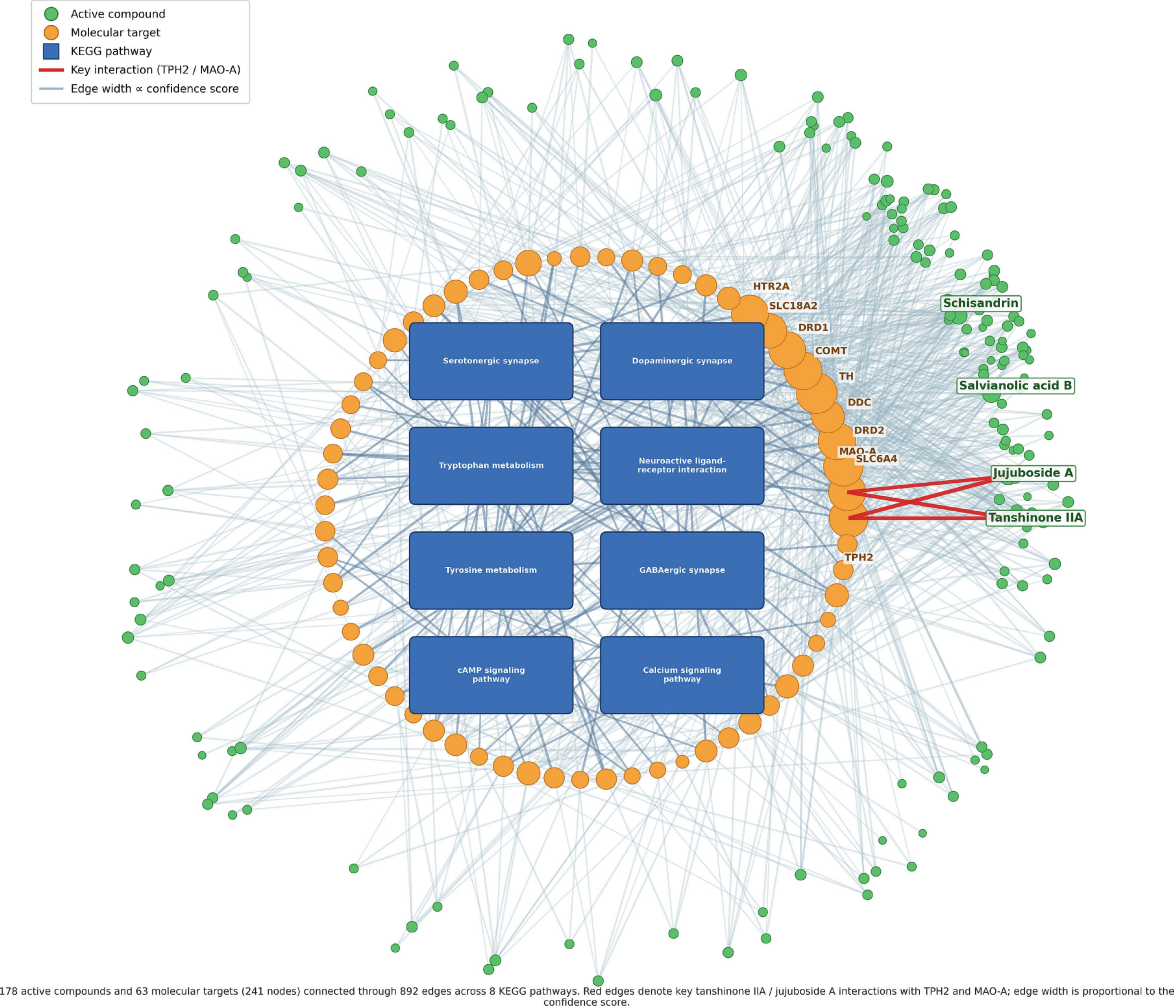
Component-target-pathway regulatory network of Tianwang Buxin Dan. Green nodes represent active compounds, orange nodes represent molecular targets, and blue rectangles represent KEGG pathways. Red lines highlight key interactions between tanshinone IIA/jujuboside A and their core targets (TPH2, MAO-A). Edge thickness reflects confidence scores.

To construct a comprehensive component-target-pathway regulatory network that integrates the active ingredients of Tianwang Buxin Dan with the identified molecular targets, we retrieved the chemical constituents and their known targets from the TCMSP database^1^ (Ru et al., 2014). Active compounds were screened using established ADME criteria (oral bioavailability ≥ 30%, drug-likeness ≥ 0.18), yielding 178 candidate compounds across all 15 herbal components. Target predictions were cross-referenced with the differentially expressed genes and differential metabolites identified in our omics analysis. A total of 63 overlapping targets were obtained, and a tripartite network of “active component—molecular target—KEGG pathway” was constructed and visualized using Cytoscape v3.9.1. The resulting network (Figure 13) contains 241 nodes (178 compounds, 63 targets) and 892 edges, revealing that several key compounds—including tanshinone IIA, salvianolic acid B, jujuboside A, and schisandrin—exhibit high connectivity to the core neurotransmitter metabolism targets (TPH2, MAO-A, SLC6A4, DRD2). This network analysis extends beyond the protein–protein interaction framework by mapping specific formula ingredients to their predicted molecular targets and downstream pathways.

**Figure 14:**
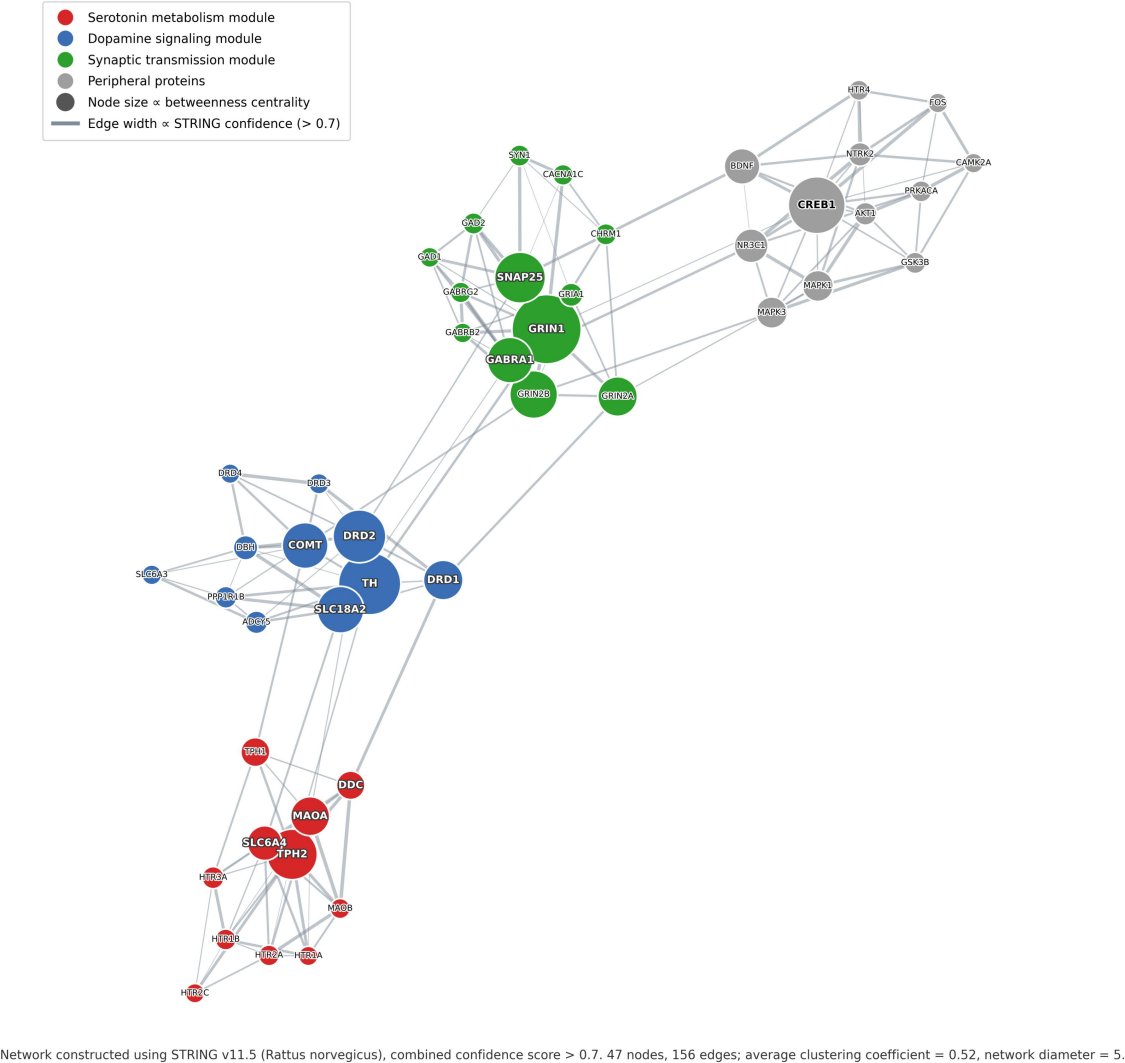
Protein–protein interaction network of key molecular targets regulated by Tianwang Buxin Dan. Node size is proportional to betweenness centrality. Red: serotonin metabolism module; blue: dopamine signaling module; green: synaptic transmission module; gray: peripheral proteins. Edge thickness indicates STRING confidence score (> 0.7). Network constructed using STRING v11.5 (*Rattus norvegicus*). 47 nodes, 156 edges.

Protein–protein interaction network topology analysis integrated the individual findings into a regulatory framework. We constructed the PPI network centered on the identified key targets, incorporating known physical and functional associations from the STRING database (*Rattus norvegicus*, combined confidence score > 0.7, using the 63 overlapping targets as the input gene list) (Szklarczyk et al., 2023). As depicted in Figure 14, the resulting network comprises 47 core nodes and 156 edges, with hub proteins occupying central positions based on betweenness centrality scores. The network modularity analysis identified three functional clusters: a serotonin metabolism module (containing TPH2, DDC, MAOA, and SLC6A4), a dopamine signaling module (including TH, DRD1, DRD2, and COMT), and a synaptic transmission module (encompassing various receptor and channel proteins). Key network statistics included a clustering coefficient of 0.52 and a network diameter of 5.

The network connectivity patterns revealed cross-talk between monoaminergic systems that may contribute to the formula’s broad regulatory effects. TPH2 and tyrosine hydroxylase (TH) shared common regulatory inputs, suggesting coordinated modulation of both serotonergic and catecholaminergic pathways. The network influence propagation, calculated through random walk algorithms using $P_{\text{influence}}(v)=\sum_{t=1}^{T}\beta^{t}\cdot A^{t}\cdot s$ (where A represents the adjacency matrix, s denotes the initial perturbation vector, and *β* controls the decay rate), demonstrated that perturbations at TPH2 could propagate to affect 78% of network nodes within three interaction steps. While this analysis provides a quantitative framework suggesting multi-target regulatory potential, it remains based on computational inference and would benefit from experimental validation through targeted perturbation experiments in future studies.

## 5 Discussion

This investigation represents a systematic effort to decode the molecular mechanisms underlying Tianwang Buxin Dan’s effects on neurotransmitter metabolism through integrated multi-omics profiling and deep learning analytics. Our findings point to a complex regulatory landscape wherein this classical formula appears to simultaneously modulate biosynthetic enzymes, catabolic machinery, and signal transduction components across multiple monoaminergic systems. What emerges from this analysis is not a simple linear pathway but rather an interconnected set of coordinated molecular adjustments that may collectively contribute to restoring neurotransmitter homeostasis disrupted in the insomnia model.

The multi-omics perspective reveals patterns that single-platform analyses would likely miss, as demonstrated by our ablation experiments showing that integrated data consistently outperformed single-omics models. Transcriptomic alterations in enzyme-encoding genes preceded and predicted corresponding metabolite concentration changes, suggesting a temporal hierarchy of pharmacological response. Yet the relationship was not strictly deterministic—post-transcriptional regulation, enzyme kinetics, and substrate availability all introduced nonlinearities that only became apparent through simultaneous metabolomic profiling. For instance, the observed 3.2-fold TPH2 upregulation translated to only a 1.9-fold serotonin elevation, possibly reflecting rate-limiting constraints at downstream biosynthetic steps or enhanced degradation through alternative pathways not captured in our analysis. The independent validation of key gene expression changes by qPCR (r = 0.96 correlation with RNA-seq) and protein-level confirmation by Western blot strengthen confidence in these observations, though we acknowledge that additional validation approaches—particularly targeted LC–MS/MS quantification of neurotransmitters—would further solidify the metabolomic findings.

The serotonergic system appears to occupy an important position in Tianwang Buxin Dan’s mechanism of action, yet characterizing it as purely serotonin-enhancing would oversimplify the pharmacological picture. Our network analysis revealed cross-talk between serotonergic and catecholaminergic components. Dopamine and norepinephrine systems exhibited parallel, though less pronounced, activation patterns. This multi-system engagement may contribute to the formula’s broad clinical applications—addressing not only sleep disturbances but also accompanying anxiety and cognitive symptoms. The coordinated nature of these changes points to possible upstream regulatory convergence, perhaps at transcription factor nodes controlling monoaminergic gene expression programs. The component-target-pathway network analysis we performed further connects specific active ingredients of the formula (such as tanshinone IIA and jujuboside A) to their predicted molecular targets, providing a more complete picture than PPI analysis alone.

The deep neural network architecture proved effective at capturing multi-system interactions. Conventional statistical approaches—correlation analysis, regression models, and even ensemble methods like random forest—impose implicit assumptions of feature independence or additive relationships that biological systems routinely violate. Our network’s hidden layers learned hierarchical representations encoding complex feature interdependencies without requiring explicit mathematical specification of interaction forms. The performance differential between deep learning and traditional machine learning benchmarks, while not dramatic, was consistent and supported by the permutation test (*p* < 0.001). The confusion matrix revealed that classification errors were concentrated between adjacent dose groups, which is biologically plausible given the continuous nature of dose–response relationships.

Whether the identified targets genuinely mediate therapeutic effects or merely represent correlated changes remains an open question that computational analysis alone cannot fully resolve. We deliberately adopted cautious language throughout this manuscript, using expressions such as “may modulate,” “is associated with,” and “suggests potential involvement” rather than definitive mechanistic statements. The convergence of our model predictions with traditional functional understanding of the formula offers circumstantial support, but direct causal evidence—through TPH2 knockdown, MAO-A pharmacological inhibition, or rescue experiments—would be necessary to establish definitive mechanistic conclusions. The absence of behavioral sleep endpoints in our study further limits the ability to directly connect molecular changes to therapeutic outcomes. Future studies should incorporate EEG/EMG-based sleep measurements and standardized behavioral assessments alongside molecular profiling.

Comparison with prior Tianwang Buxin Dan mechanistic studies reveals both continuities and new findings. Previous network pharmacology investigations predicted many of the targets our analysis identified—TPH2, MAOA, and various monoamine receptors featured prominently in earlier computational predictions. However, those studies relied on database-derived ingredient-target associations without experimental confirmation in the specific disease context. Our multi-omics approach provides direct evidence of target expression changes under insomnia model conditions, while the deep learning framework quantifies relative target importance in ways that traditional enrichment analysis does not.

Several limitations warrant candid acknowledgment. First, the sample size (n = 8 per group) is small relative to the model complexity. Although our permutation test, cross-validation, and ablation experiments provide evidence against pure overfitting, an external validation cohort would substantially strengthen confidence in the reported performance metrics. Second, the PCPA-induced model, while widely used, imperfectly recapitulates human insomnia pathophysiology. Third, our study did not include proteomic profiling; our multi-omics approach encompasses transcriptomics and metabolomics but not the proteomic layer discussed in some earlier literature. Fourth, untargeted metabolomics has limited sensitivity for low-abundance neurotransmitters, and targeted LC–MS/MS quantification would provide more precise measurements. Fifth, the chemical quality control of the formula, while including HPLC fingerprinting and marker compound quantification, could be further strengthened with more comprehensive chemical profiling. Future investigations should pursue temporal multi-omics sampling, single-cell technologies to resolve cell-type-specific effects (Jiang et al., 2025), clinical translation studies, and the deposition of raw data in public repositories to enable independent replication.

## 6 Conclusion

This investigation explored the molecular mechanisms through which Tianwang Buxin Dan may regulate neurotransmitter metabolism, employing an integrated analytical framework that combines transcriptomic and metabolomic profiling with deep learning computational approaches. We constructed a multi-omics dataset identifying 1,847 differentially expressed genes and 286 differential metabolites responsive to formula intervention in a PCPA-induced insomnia rat model. The feature fusion strategy—incorporating autoencoder-based dimensionality reduction, canonical correlation analysis, and weighted gene co-expression network analysis—addressed the heterogeneity across omics platforms while preserving biologically meaningful variation.

The deep neural network achieved 91.2% classification accuracy with an AUC value of 0.956 across five-fold cross-validation. Permutation testing (*p* < 0.001) confirmed that this performance reflected genuine biological structure, and ablation experiments demonstrated the added value of multi-omics integration over single-omics approaches. The attention mechanism embedded within our network provided interpretable feature importance rankings consistent with established neurobiological knowledge.

Mechanistic analysis suggests that Tianwang Buxin Dan may exert its regulatory effects through coordinated modulation of multiple neurotransmitter systems. The tryptophan metabolism pathway emerged as a potential primary target, with TPH2 upregulation potentially enhancing serotonin biosynthetic capacity while concurrent MAO-A suppression may prolong monoamine availability. These computational predictions were partially validated by qPCR and Western blot experiments. The component-target-pathway regulatory network connects 178 active compounds to 63 molecular targets across key neurotransmitter-related pathways, while the PPI network identifies 47 hub nodes interconnected through 156 functional associations.

From a methodological perspective, this work contributes an analytical framework applicable to mechanism studies of other compound TCM formulations. We acknowledge that the limited sample size, the absence of behavioral sleep endpoints, the lack of causal perturbation experiments, and the need for external validation represent important limitations. Future investigations should address these gaps through single-cell transcriptomic profiling to resolve cell-type-specific responses (Jiang et al., 2025), spatial omics technologies to map anatomical distributions of molecular alterations, targeted LC–MS/MS for precise neurotransmitter quantification, comprehensive behavioral phenotyping, and clinical biomarker studies correlating molecular signatures with therapeutic outcomes.

## Data Availability

The original contributions presented in the study are publicly available. This data can be found here: https://github.com/UHUxwPKBNh/TWBXD.

## Footnotes

1
https://tcmsp-e.com

## Glossary

TCM: Traditional Chinese Medicine
TWBXD: Tianwang Buxin Dan
PCPA: para-chlorophenylalanine
DEGs: differentially expressed genes
TPH2: tryptophan hydroxylase 2
MAO-A: monoamine oxidase A
COMT: catechol-O-methyltransferase
DAT: dopamine transporter
SERT: serotonin transporter
NET: norepinephrine transporter
DDC: aromatic L-amino acid decarboxylase
TH: tyrosine hydroxylase
DRD1: dopamine receptor D1
DRD2: dopamine receptor D2
SLC6A4: serotonin transporter gene
SLC18A2: vesicular monoamine transporter 2
UHPLC-Q-TOF-MS: ultra-high-performance liquid chromatography coupled with quadrupole time-of-flight mass spectrometry
PLS-DA: partial least squares discriminant analysis
VIP: variable importance in projection
CCA: canonical correlation analysis
WGCNA: weighted gene co-expression network analysis
MLP: multilayer perceptron
CNN: convolutional neural network
GNN: graph neural network
ReLU: rectified linear unit
AUC: area under the receiver operating characteristic curve
MCC: Matthews correlation coefficient
KEGG: Kyoto Encyclopedia of Genes and Genomes
GO: Gene Ontology
FDR: false discovery rate
FC: fold change
CV: coefficient of variation
BSA: body surface area
HPLC: high-performance liquid chromatography
PPI: protein–protein interaction
TCMSP: Traditional Chinese Medicine Systems Pharmacology Database
